# Multi-locus sequence analyses reveal a clonal *L. borgpetersenii* genotype in a heterogeneous invasive *Rattus* spp. community across the City of Johannesburg, South Africa

**DOI:** 10.1186/s13071-020-04444-0

**Published:** 2020-11-11

**Authors:** Mark Moseley, Kovashnee Naidoo, Armanda Bastos, Liezl Retief, John Frean, Sandra Telfer, Jennifer Rossouw

**Affiliations:** 1 School of Biological Sciences, University of Aberdeen, Aberdeen, UK; 2 National Institute for Communicable Diseases, Division of National Health Laboratory Service, Johannesburg, South Africa; 3 Department of Zoology and Entomology, University of Pretoria, Pretoria, South Africa; 4 Wits Research Institute for Malaria, University of the Witwatersrand, Johannesburg, South Africa

**Keywords:** Leptospirosis, Zoonosis, *lfb1*, Urbanisation, Rats, Public health, Molecular epidemiology, Disease ecology

## Abstract

**Background:**

*Rattus* spp. are frequently implicated as key reservoir hosts for leptospirosis, one of the most common, but neglected, bacterial zoonoses in the world. Although leptospirosis is predicted to be a significant public health threat in Africa, studies from the continent are limited.

**Methods:**

*Rattus* spp. (*n* = 171) were sampled (January–May 2016) across the City of Johannesburg, South Africa’s largest inland metropole. *Rattus* spp. genetic diversity was evaluated by full length (1140 bp) cyt *b* sequencing of 42 samples. For comparison, a further 12 *Rattus norvegicus* samples collected in Cape Town, South Africa’s largest coastal metropole, were also genotyped. *Leptospira* infections were identified and genotyped using real-time PCR and multi-locus (*lfb1*, *secY* and *lipL41*) DNA sequencing.

**Results:**

Five *R. norvegicus* haplotypes were identified across Johannesburg, four of which have not previously been detected in South Africa, and one in Cape Town. Across Johannesburg we identified a *Leptospira* spp. infection prevalence of 44% (75/171) and noted significant differences in the prevalence between administrative regions within the metropole. Multi-locus sequence analyses identified a clonal genotype consistent with *L. borgpetersenii* serogroup Javanica (serovar Ceylonica).

**Discussion:**

The prevalence of infection identified in this study is amongst the highest detected in *Rattus* spp*.* in similar contexts across Africa. Despite the complex invasion history suggested by the heterogeneity in *R. norvegicus* haplotypes identified in Johannesburg, a single *L. borgpetersenii* genotype was identified in all infected rodents. The lack of *L. interrogans* in a rodent community dominated by *R. norvegicus* is notable, given the widely recognised host-pathogen association between these species and evidence for *L. interrogans* infection in *R. norvegicus* in Cape Town. It is likely that environmental conditions (cold, dry winters) in Johannesburg may limit the transmission of *L. interrogans*. Spatial heterogeneity in prevalence suggest that local factors, such as land use, influence disease risk in the metropole.

**Conclusions:**

In South Africa, as in other African countries, leptospirosis is likely underdiagnosed. The high prevalence of infection in urban rodents in Johannesburg suggest that further work is urgently needed to understand the potential public health risk posed by this neglected zoonotic pathogen.
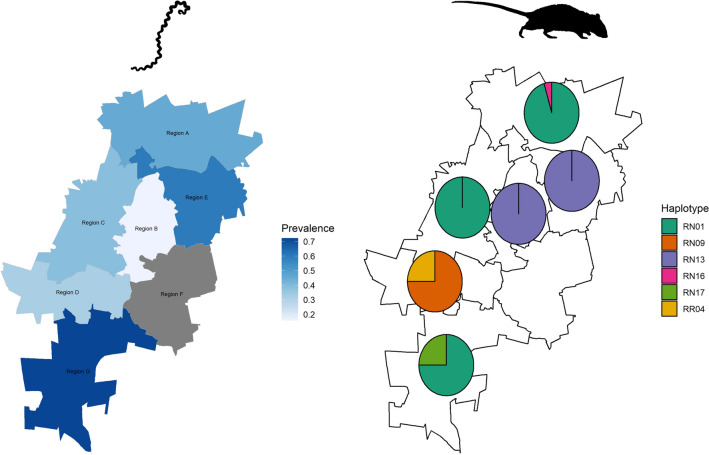

## Background

Leptospirosis is one of the world’s most common, but neglected, zoonotic diseases. Globally, there are an estimated 1 million cases and 60,000 deaths annually [1] with a disproportionate burden of the disease falling on low-income communities in the developing world [2]. The majority of human cases of leptospirosis are as a result of infection with three pathogenic species, *Leptospira interrogans*, *Leptospira borgpetersenii* and *Leptospira kirschneri* [3]. Serologically, *Leptospira* are divided into over 300 serovars with antigenically-related serovars clustered into serogroups [3]. *Rattus norvegicus* are traditionally associated with *L. interrogans* serogroup Icterohaemorrhagiae [3], and generally have a higher prevalence of infection than other *Rattus* spp. [4]. Exposure to this key urban rodent pest has been demonstrated to be associated with increased risk of leptospirosis [5].

Regions of Africa are predicted to have some of the highest leptospirosis disease burdens in the world, although estimates are uncertain due to a lack of studies from the continent [1, 2]. Where surveillance in rat populations has been undertaken in Africa, the prevalence of infection is highly variable (range 0–68%) [4]. In South Africa, genetic analyses have identified *R. norvegicus*, *R. rattus* and *R. tanezumi* in urban environments [6] and *R. norvegicus* has been implicated in an outbreak of leptospirosis in a correctional facility in Cape Town [7].

Although recent surveillance studies have identified *Leptospira* spp. infection in cattle [8] and horses [9] in South Africa, there are no recent published surveillance data describing the prevalence and diversity of *Leptospira* infections in rats in urban environments. Therefore, we aimed to (i) identify the prevalence of *Leptospira* spp. infection in urban rats in Johannesburg, South Africa’s largest inland metropole, (ii) characterise the genetic diversity of both *Leptospira* spp. infections and their rodent hosts across the metropole, and (iii) contrast these results to previously identified *Leptospira* infections [7] and *R. norvegicus* hosts from Cape Town, South Africa’s largest coastal metropole.

## Methods

### Sample selection and *Rattus* spp. typing

**Fig. 1:**
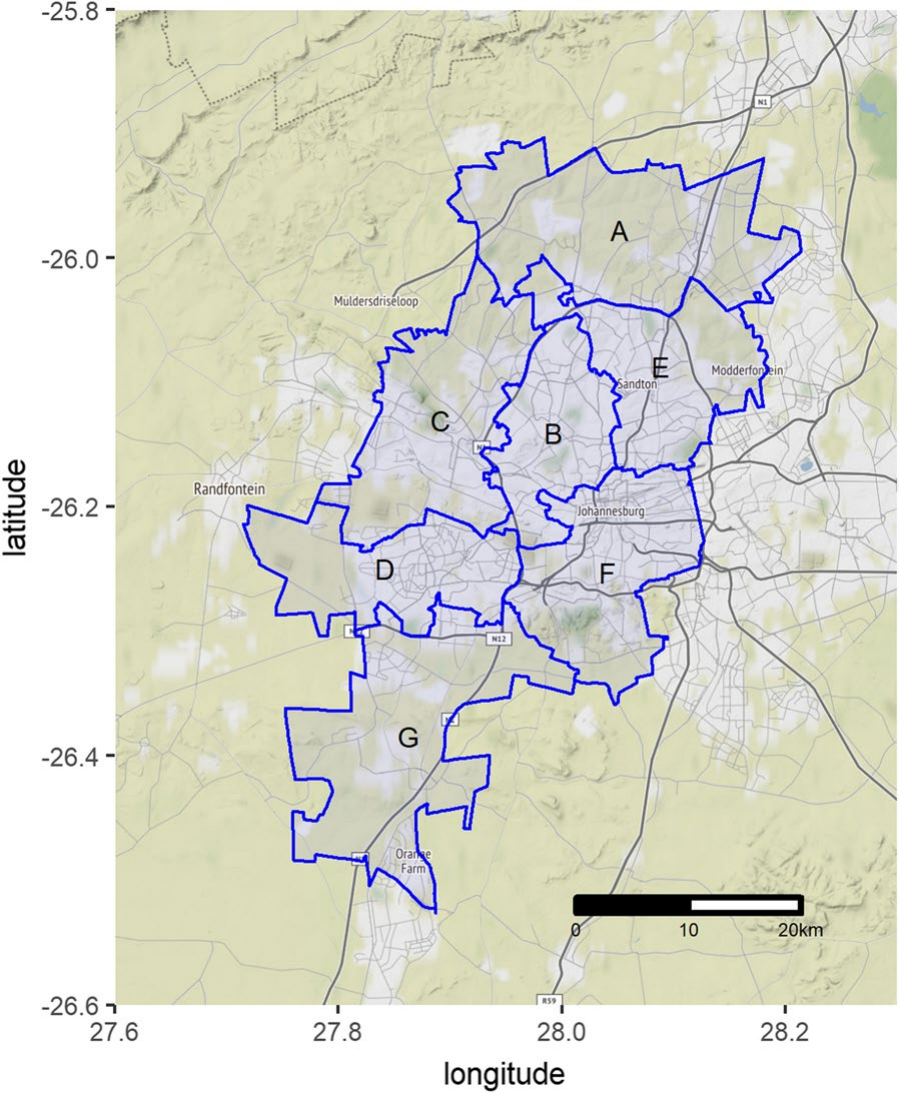
The seven administrative regions (A–G) of the City of Johannesburg metropolitan municipality

We sampled 171 *Rattus* spp. collected across the 7 administrative regions of the City of Johannesburg municipality (Fig. 1) as part of a plague (*Yersinia pestis*) surveillance program between January and May 2016. Johannesburg is an inland, high-elevation (~1700 m) site that is characterised by cold, dry winters and warm, wet summers (Additional file 1: Figure S1). Invasive *Rattus* spp. were presumptively identified based on morphology (weight and tail to body length ratio). *Rattus* spp.  > 250 g and with tail/body < 1 were classified as *R. norvegicus*. Animals with equivocal morphological measurements (*n* = 32) and a subset (*n* = 10) of *R. norvegicus* with unequivocal morphological measurements were further genotyped by cyt *b* gene characterisation as previously described [6]. DNA extractions were performed on kidney samples using the QIAamp DNA Mini Kit on a QIAcube system (Qiagen, Hilden, Germany) according to manufacturer’s instructions. For comparison, *R. norvegicus* samples (*n* = 12) collected during an outbreak of human leptospirosis in a correctional facility in Cape Town [7] also underwent cyt *b* characterisation. Cytochrome *b* PCR products were purified as previously described [6] and sequenced at the core Sanger sequencing facility at the University of Pretoria.

### *Leptospira* spp. detection and prevalence estimation

*Leptospira* spp. infections were detected using a diagnostic real-time PCR targeting 300 bp of the *lfb1* gene and incorporating melt curve analysis to identify infecting *Leptospira* species [10]. As the standard *lfb1* primers may underestimate the prevalence of *L. borgpetersenii* infections and *L. interrogans-L. borgpetersenii* mixed infections may occur [11], samples which initially tested negative, as well as samples (*n* = 12) from Cape Town, 8 of which had previously been identified as infected with *L. interrogans* [7], were retested using a *L. borgpetersenii* species-specific forward primer [11]. All real-time PCR assays were performed as previously described [7, 11]. Prevalence estimates and logit confidence intervals were estimated using the *binom* package (https://cran.r-project.org/package=binom) and mapped using the *ggmap* package (https://cran.r-project.org/package=ggmap). The prevalence estimates from the six regions in which more than 10 animals were sampled were compared using Chi-square tests. All analyses were performed in R version 3.6.1.

### Genotyping of *Leptospira* spp. infections

To confirm the *Leptospira* species classification based on melt curve analysis [10], we sequenced a subset (*n* = 37) of the *lfb1* amplicons. Although the *lfb1* locus has been demonstrated to provide valuable phylogenetic data [12], these initial sequences from samples collected across five regions revealed no sequence polymorphisms. Therefore, we subsequently sequenced additional loci, *secY* (~450 bp) (*n* = 13) and *lipL41* (~500 bp) (*n* = 5), from a subset of samples to increase the resolution of the molecular typing and allow identification of the presumptive serogroup. Similarly, infections in *R. norvegicus* in Cape Town previously identified as *L. interrogans* by sequencing of the *lfb1* amplicon [7] were further typed by sequencing *secY* (*n* = 3) and intergenic regions MST1, MST3 and MST9 (*n* = 3) [13] to determine whether further genetic resolution was possible. Primer pairs secYFd/secYR3 and lipL41F3/lipL41R3 were used to amplify *secY* and *lipL41* [14] and MST1, MST3 and MST9 were amplified using published primers [13] on a Techne TC5000 system (Techne Inc., Burlington, USA). The total reaction volume of 25 µl consisted of 5 µl of DNA extraction, primer concentrations of 0.5 µM, 12.5 µl of MyTaq red mix 2× (Bioline Reagents Ltd., London, UK) and 5.5 µl of molecular grade water. A “touchdown” thermal profile comprising initial denaturation at 95 °C for 3 min, followed by 40 cycles of denaturation at 95 °C for 20 s, variable annealing for 25 s and extension at 72 °C for 40 s, with a final extension at 72 °C for 7 min was performed. The annealing temperature was reduced from 60 °C to 50 °C over the first 10 cycles and then maintained at 46 °C. Each PCR run included a negative control (molecular grade water) for every 4 samples, and a positive control (*L. borgpetersenii* strain 201501056 for *L. borgpetersenii*-specific assays and *L. interrogans* strain 201501067 for all other assays). *Leptospira* PCR products were purified using the QIAquick PCR Purification Kit (Qiagen) according to manufacturer’s instructions, with a final elution in 35 µl. The purified product was quantified using a Nanodrop ND1000 spectrophotometer (Thermo Fisher Scientific, Waltham, USA) and sequenced by Eurofins Genomics GmbH (Ebersburg, Germany).

### Phylogenetic analyses of *Rattus* spp. cytochrome *b* sequences and *Leptospira* spp. multi-locus sequences

Sequences for each locus were aligned using the ClustalW algorithm and the most appropriate evolutionary model determined using MEGA7 [15]. Cytochrome *b* sequences from previous studies of *Rattus* spp. in South Africa [6] were used as reference sequences. *Leptospira* reference sequences were obtained by querying sequences against the NCBI refseq_genome database using the BLASTn algorithm limited to *Leptospira* (taxid 171) belonging to the two species (*L. interrogans* and *L. borgpetersenii*) identified in this study. Aligned BLAST hits for each locus were linked by NCBI Biosample accession and representative sequences for each *Leptospira* species and serovar combination selected as reference sequences.

To characterise *Leptospira* spp. genetic diversity, multi-locus phylogenetic analyses were implemented in BEAST v2.6.0 [16] using each locus as a separate partition with unlinked substitution models and linked clocks (strict) and trees. The most appropriate substitution models as determined by model test in MEGA7 [15] were used for each locus. Multi-locus analyses were run using a chain length of 1 × 10^7^ and sampled every 1 × 10^3^ runs with a ‘burn-in’ of 10%. TRACER v1.7.1 [17] was used to verify that the effective sample size (ESS) was greater than 200 and TREEANNOTATOR v2.6.0 was used to generate a maximum clade credibility tree using mean node heights annotated by posterior probabilities greater than 0.9. Trees were annotated using the R package *ggtree* [18].

## Results

### *Rattus* spp. genetic typing and distribution

**Fig. 2:**
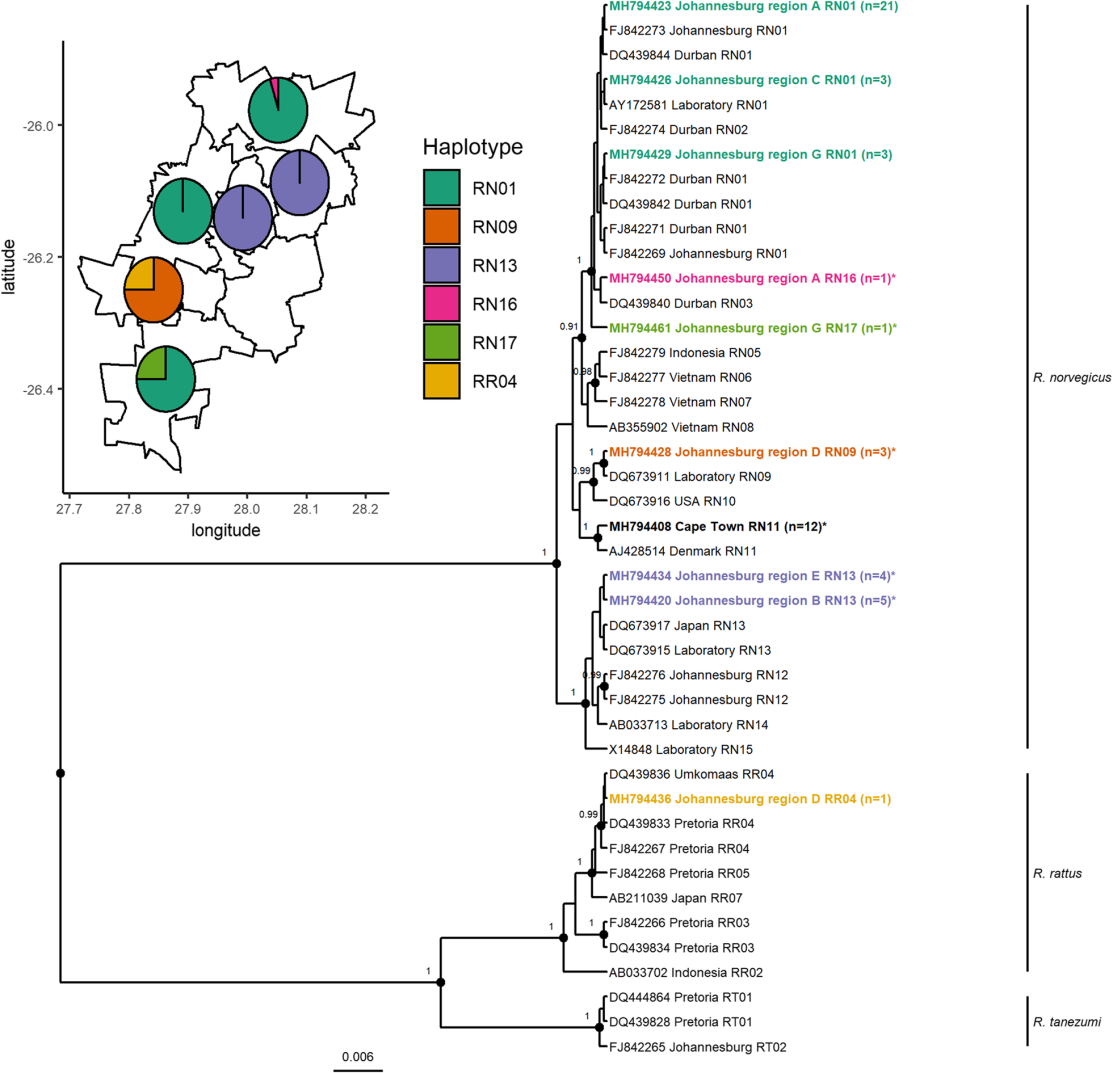
The *Rattus* spp. haplotypes identified in Cape Town and Johannesburg and the distribution of haplotypes across six administrative regions of the City of Johannesburg (inset). Haplotypes identified in this study are shown in bold and number of animals are indicated in parentheses. Haplotypes not previously detected in South Africa are indicated with a *. The phylogeny is based on analysis of full-length (1140 bp) cyt *b* sequences utilising the Hasegawa-Kishino-Yano evolutionary model [19]. Nodes with posterior support greater than 0.9 are labelled

In Johannesburg, cyt *b* typing identified 98% (41/42) of the typed rodent samples as *R. norvegicus* and one (with equivocal morphological measurements) as *R. rattus* (Fig. 2). The majority of *R. norvegicus* from Johannesburg clustered with the RN01 (*n* = 27), the only *R. norvegicus* haplotype identified in this study that has previously been detected in South Africa [6]. The second most common haplotype, RN13 (*n* = 9), is associated with laboratory *R. norvegicus* strains and animals captured in Japan. These two haplotypes demonstrated distinct geographical associations within the Johannesburg metropole, with RN01 restricted to regions A, C and G and RN13 restricted to regions B and E (Fig. 2, inset). Two further samples were identified as two new haplotypes (RN16 and RN17), closely related to RN01 and RN03. The remaining samples (*n* = 3) clustered with a laboratory strain haplotype (RN09). The single *R. rattus* sample clustered with RR04, a haplotype previously detected in South Africa [6]. All *R. norvegicus* from Cape Town shared a haplotype associated with rats from Denmark (RN11). This represents the first record of this haplotype in South Africa.

### *Leptospira* spp. prevalence and genetic diversity

**Fig. 3:**
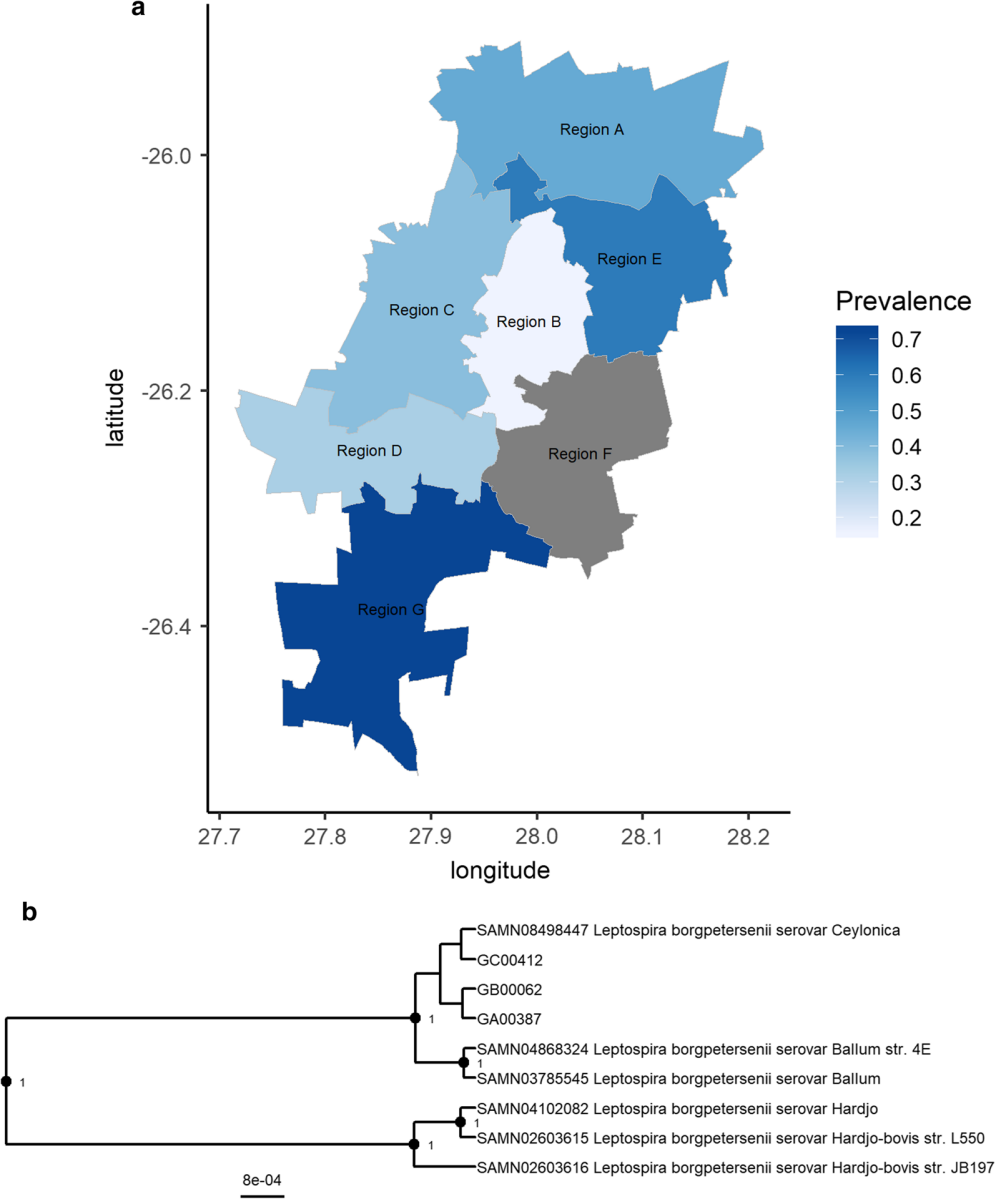
**a** The prevalence of *Leptospira* sp. infection in *Rattus* spp. in the six administrative regions of the City of Johannesburg in which more than 10 animals were sampled. Region F, where three animals were sampled, is shaded in grey. All infections were typed by *lfb1* melt curve analysis as *L. borgpetersenii*. **b** Bayesian multi-locus (*lfb1*, 167 bp; *secY*, 431 bp; and *lipL41*, 592 bp) phylogeny of *L. borgpetersenii* detected in three samples from three administrative regions (A, B and C) of Johannesburg. Nodes with posterior support greater than 0.9 are labelled. Reference sequences are labelled by biosample and *Leptospira* strain. Individual gene trees including all samples successfully typed for each locus are found in Additional file 3: Figures S3–S5

Across Johannesburg, *Leptospira* was detected in 44% (75/171) of samples and in 86% (6/7) of the metropole’s administrative regions (Fig. 3a). In the region (F) where no infection was detected, only 3 animals were sampled. In the remaining six regions, in which between 18 and 53 animals were sampled, prevalence ranged from 16–72% (Additional file 2: Table S1 and Figure S2) and the difference in prevalence was significant (*χ*^2^ = 16.68, *df* = 5, *P* = 0.005). Melt curve analysis confirmed *L. borgpetersenii* in all positive samples, including that from the only *R. rattus* identified. Although the standard *lfb1* primers [10] identified 81% (61/75) of positive samples, the remaining 14 *L. borgpetersenii*-positive samples were only identified using the *L. borgpetersenii*-specific forward primer [11]. Notably, no animals were found to be infected with *L. interrogans* in Johannesburg. Multi-locus phylogenetic analysis based on sequencing *lfb1*, *secY* and *lipL41* confirmed the presence of a single genotype of *L. borgpetersenii* identical to strains belonging to serovar Ceylonica (serogroup Javanica) (Fig. 3b).

**Fig. 4:**
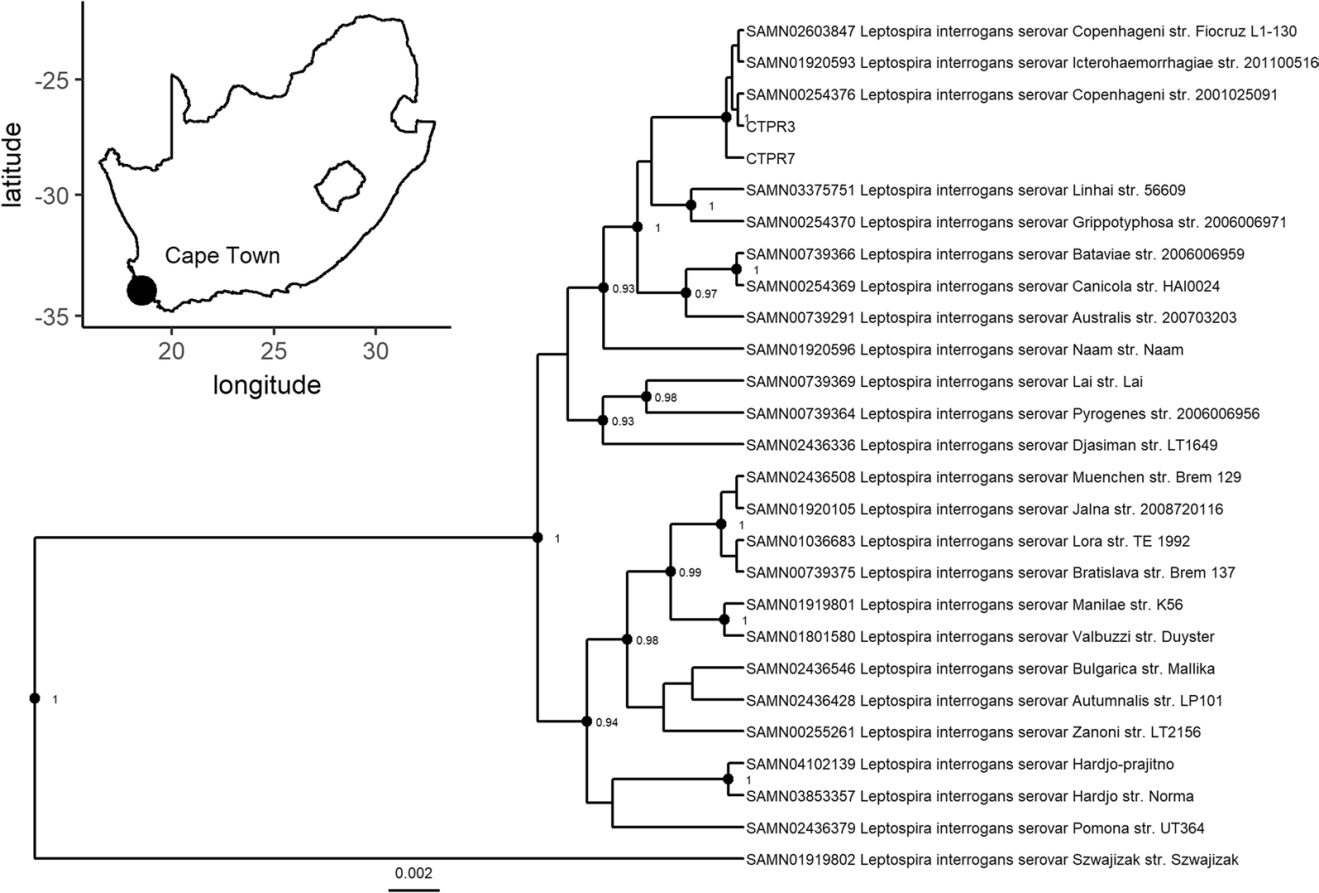
Bayesian multi-locus (*lfb1*, 261 bp; *secY*, 433 bp; MST1, 174 bp; MST3, 220 bp; and MST9, 204 bp) phylogeny, implemented with unlinked substitution models, of *L. interrogans* strains identified in Cape Town (inset). Nodes with posterior support greater than 0.9 are labelled. Reference sequences are labelled by biosample and *Leptospira* strain. Individual gene trees including all samples successfully typed for each locus are found in Additional file 3: Figures S6–S10

Further molecular typing and phylogenetic analysis of the *L. interrogans* infections identified in *R. norvegicus* in Cape Town (Fig. 4) confirmed previous results, based on *lfb1* sequencing, that identified a single genotype consistent with strains belonging to serovars Copenhagenii/Icterohaemorrhagiae (serogroup Icterohaemorrhagiae) [7]. However, application of the *L. borgpetersenii*-specific *lfb1* assay to these samples revealed the presence of a single *L. interrogans-L. borgpetersenii* mixed infection with the *L. borgpetersenii lfb1* sequence identical to those detected in Johannesburg (Additional file 3: Figure S3).

## Discussion

We identified a high prevalence of infection with a clonal *L. borgpetersenii* genotype in a diverse urban *R. norvegicus* community in Johannesburg, South Africa’s largest metropole. The spatial structuring exhibited by the two most common *R. norvegicus* haplotypes and evidence for the presence of at least four haplotypes not previously detected in South Africa suggest that the *R. norvegicus* community in Johannesburg is a result of multiple invasion events. The most common haplotype (RN01), along with two new haplotypes (RN16 and RN17), was genetically similar to haplotypes previously identified in Johannesburg and Durban [6], a coastal city on South Africa’s east coast. However, all the haplotypes identified in Johannesburg were distinct from the single haplotype identified in Cape Town. Further *Rattus* spp. genotyping, both within Johannesburg and nationally, would be valuable to better understand the invasion history of these key hosts.

Despite the heterogenous *R. norvegicus* community identified in Johannesburg, we identified a single clonal *Leptospira* genotype consistent with *L. borgpetersenii* serogroup Javanica in *R. norvegicus* across the metropole and in the only *R. rattus* identified in this study. Although clonality is inferred based on sequencing a limited number of loci, for each of these phylogenetically informative loci [12] multiple samples were sequenced and no genetic variation was noted. A similar lack of genetic diversity in *L. borgpetersenii* serogroup Javanica strains in *Rattus* spp. hosts has been noted across Malaysia using pulsed-field gel electrophoresis (PFGE), the gold standard for genotyping of *Leptospira* [20]. Moreover, the *lfb1* sequence from a single *L. borgpetersenii* infection identified as part of a mixed infection in Cape Town was also consistent with *L. borgpetersenii lfb1* sequences identified in Johannesburg suggesting that, as in Malaysia [20], this strain may be widely distributed in South Africa. However, as this infection was identified as part of a mixed infection, further genotyping at additional loci was not possible.

The lack of *L. interrogans* in *Rattus* spp. in Johannesburg is notable, given the widely recognised host-pathogen association between *Rattus* spp. and serogroups in this species [21] and recent evidence that *L. interrogans* is found in *R. norvegicus* in Cape Town [7]. It is possible that, despite the complex invasion history suggested by *R. norvegicus* haplotyping, none of the *R. norvegicus* introduced to Johannesburg were infected with *L. interrogans*. However, even where studies have demonstrated significant levels of *L. borgpetersenii* infection in *Rattus* spp., *L. interrogans* was the more commonly detected species [20]. *Leptospira interrogans* and *L. borgpetersenii* differ in their ability to survive in the environment [22] and field studies in rodent hosts suggest that warm, moist environments favour the transmission of *L. interrogans* while environmental conditions are less important for the transmission of *L. borgpetersenii* [23, 24]. Therefore, it is more likely that the environmental conditions in Johannesburg (cold, dry winters) explains the absence of *L. interrogans,* whereas environmental conditions in Cape Town (coastal, temperate conditions with wet winters and warm summers) allow the transmission of both *Leptospira* species.

Within Johannesburg, there were significant differences in the prevalence of infection between administrative regions, consistent with previous findings of high variance in infection prevalence in urban *R. norvegicus* in Copenhagen, Denmark [25]. Previous studies have suggested a link between increased *L. borgpetersenii* prevalence in rodent hosts and urban areas characterised by mixed residential and commercial use in Malaysian Borneo [23]. In this study, the region with the lowest prevalence (region B) includes upmarket residential areas and business districts with less habitat heterogeneity than other regions, which include a mix of residential, business and industrial areas, large informal settlements and semi-rural areas. In this study, spatial resolution below the level of regions was not possible. Therefore, further work is required to identify the drivers of spatial variation in *Leptospira* prevalence in *Rattus* spp. in urban areas in South Africa.

Although the prevalence of *Leptospira* spp. infection in *Rattus* spp. varies widely in Africa [4], the prevalence (44%, 75/171) identified here, and in a previous study undertaken in Cape Town [7], are amongst the highest identified in similar contexts in the region. For example, the prevalence noted in this surveillance study is similar to that identified in surveillance-based sampling undertaken in urban Antananarivo in Madagascar (49%, 47/96) [26], while the prevalence (67%, 8/12) identified in *R. norvegicus* during an outbreak in Cape Town [7] is similar to that identified during an outbreak investigation (68%, 17/25) undertaken in Reunion Island [27].

## Conclusions

In South Africa, as in other African countries, leptospirosis is likely underdiagnosed and the public health risk attributable to leptospirosis in South Africa may be as high as in any other country in the region. The presumptive serogroup identified in this study (*L. borgpetersenii* serogroup Javanica) has been implicated in human leptospirosis across the Asia Pacific region [28, 29]. Therefore, identification and typing of human infections in Johannesburg is urgently needed to determine whether this serogroup is implicated in human cases of leptospirosis in South Africa’s largest metropole. Moreover, as transmission [22–24] and virulence [3] may differ between *L. interrogans* and *L. borgpetersenii,* the contrast between the *L. borgpetersenii*-dominated system in Johannesburg and other sites, such as Cape Town, represents a unique opportunity for understanding the differences in the ecology and epidemiology of these important pathogenic *Leptospira* species.

## Supplementary information

**Additional file 1: Figure S1.** The Köppen-Geiger climate classification zones for South Africa at 0.0083° resolution. Johannesburg falls within the Cwb bioclimatic zone, characterised by dry, cold winters and warm, wet summers and Cape Town falls within the Csb zone, characterised by wet winters and warm summers.

**Additional file 2: Table S1.** The prevalence of *Leptospira* infection in *Rattus* spp. in the seven administrative regions of the City of Johannesburg and in *R. norvegicus* tested during a leptospirosis outbreak investigation in the City of Cape Town. **Figure S2**. The spatial distribution of regional prevalence across the seven administrative regions of the City of Johannesburg. Region F, where only three animals were tested is excluded from the analysis

**Additional file 3: Figure S3.** Maximum clade credibility tree based on *L. borgpetersenii lfb1* sequences (167 bp) implemented using the Jukes-Cantor evolutionary model. **Figure S4.** Maximum clade credibility tree based on *L. borgpetersenii secY* sequences (433bp) implemented using the Jukes-Cantor evolutionary model. **Figure S5.** Maximum clade credibility tree based on *L. borgpetersenii lipL41* sequences (594 bp) implemented using the Jukes-Cantor evolutionary model. **Figure S6.** Maximum clade credibility tree based on *L. interrogans lfb1* sequences (261 bp) implemented using the Jukes-Cantor substitution model. **Figure S7.** Maximum clade credibility tree based on *L. interrogans secY* sequences (433 bp) implemented using the Hasegawa-Kishino-Yano evolutionary model (4) substitution model. **Figure S8.** Maximum clade credibility tree based on *L. interrogans* MST1 sequences (174 bp) implemented using the Hasegawa-Kishino-Yano evolutionary model (4) substitution model. **Figure S9.** Maximum clade credibility tree based on *L. interrogans* MST3 sequences (220 bp) implemented using the Hasegawa-Kishino-Yano evolutionary model (4) with a gamma distribution (4 categories). **Figure S10.** Maximum clade credibility tree based on *L. interrogans* MST9 sequences (204 bp) implemented using the Jukes-Cantor evolutionary model.

## Data Availability

Cytochrome *b* sequence data have been deposited in GenBank with accession numbers MH794408-MH794461. *Leptospira* sequence data have been deposited in the GenBank database under the accession numbers MH795453-MH795520.

## Abbreviations

PCR: polymerase chain reaction
DNA: deoxyribonucleic acid
NCBI: National Center for Biotechnology Information
