# Supplementary material for: Multi-locus sequence analyses reveal a clonal L. borgpetersenii genotype in a heterogeneous invasive Rattus spp. community across the City of Johannesburg, South Africa
Source: Parasit Vectors. 2020 Nov 11;13:570. doi: 10.1186/s13071-020-04444-0 (PMC7659165; doi:10.1186/s13071-020-04444-0)
Supplement: Supplementary file 1 — Additional file 1: Figure S1. The Köppen-Geiger climate classification zones for South Africa at 0.0083° resolution. Johannesburg falls within the Cwb bioclimatic zone, characterised by dry, cold winters and warm, wet summers and Cape Town falls within the Csb zone, characterised by wet winters and warm summers. [file 13071_2020_4444_MOESM1_ESM.docx]

**South Africa - bioclimatic zones**


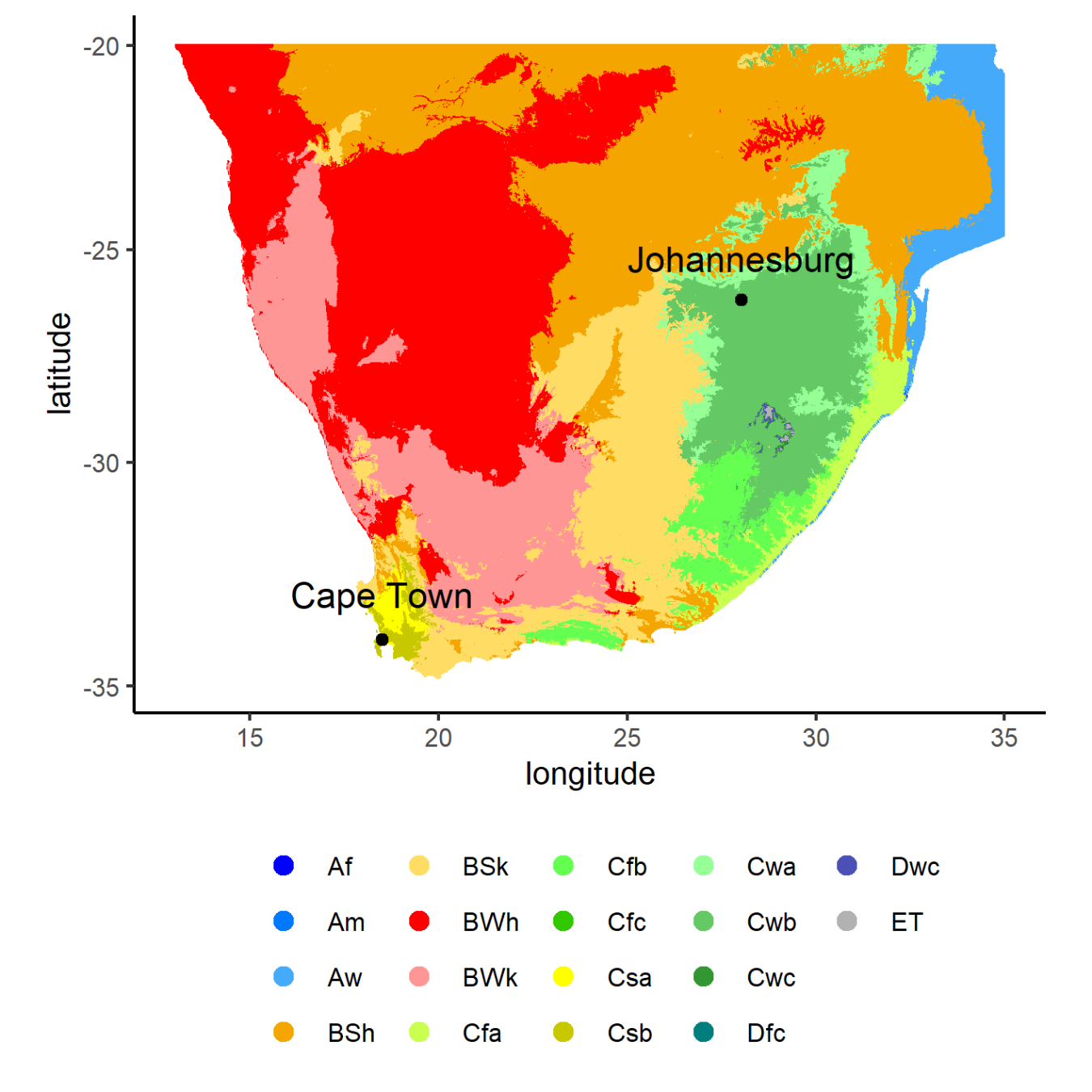


Figure S1: The Köppen-Geiger climate classification zones for South Africa at 0.0083° resolution (1). Johannesburg falls within the Cwb bioclimatic zone, characterised by dry, cold winters and warm, wet summers and Cape Town falls within the Csb zone, characterised by wet winters and warm summers.

**Description of bioclimatic zones**:

Af=Tropical, rainforest;

Am=Tropical, monsoon;

Aw=Tropical, savannah;

BSh=Arid, steppe, hot;

BSk=Arid, steppe, cold;

BWh=Arid, desert, hot;

BWk=Arid, desert, cold;

Cfa=Temperate, no dry season, hot summer;

Cfb=Temperate, no dry season, warm summer;

Cfc=Temperate, no dry season, cold summer;

Csa=Temperate, dry summer, hot;

Csb=Temperate, dry summer, warm summer;

Cwa=Temperate, dry winter, hot summer;

Cwb=Temperate, dry winter, warm summer;

Cwc=Temperate, dry winter, cold summer;

Dfc=Cold, no dry season, cold summer;

Dwc=Cold, dry winter, cold summer;

ET=Polar, tundra

Reference:

1. Beck HE, Zimmermann NE, McVicar TR, Vergopolan N, Berg A, Wood EF. Present and future köppen-geiger climate classification maps at 1-km resolution. Nat Sci Data. 2018;5:1–12.
