# Supplementary material for: Multi-locus sequence analyses reveal a clonal L. borgpetersenii genotype in a heterogeneous invasive Rattus spp. community across the City of Johannesburg, South Africa
Source: Parasit Vectors. 2020 Nov 11;13:570. doi: 10.1186/s13071-020-04444-0 (PMC7659165; doi:10.1186/s13071-020-04444-0)
Supplement: Supplementary file 2 — Additional file 2: Table S1. The prevalence of Leptospira infection in Rattus spp. in the seven administrative regions of the City of Johannesburg and in R. norvegicus tested during a leptospirosis outbreak investigation in the City of Cape Town. Figure S2. The spatial distribution of regional prevalence across the seven administrative regions of the City of Johannesburg. Region F, where only three animals were tested is excluded from the analysis [file 13071_2020_4444_MOESM2_ESM.docx]

**City of Johannesburg regional prevalence**

Table S1: The prevalence of *Leptospira* infection in *Rattus* spp. in the seven administrative regions of the City of Johannesburg and in *R. norvegicus* tested during a leptospirosis outbreak investigation (1) in the City of Cape Town.

| City  (context) | Administrative region | Regional prevalence (P/n, 95% CI) | Total prevalence (P/n, 95% CI) |
| --- | --- | --- | --- |
| Johannesburg  (surveillance) | A | 45% (24/53, 33-59%) | 44% (75/171, 37-51%) |
|  | B | 16% (3/19, 5-39%) |  |
|  | C | 38% (10/26, 22-58%) |  |
|  | D | 32% (7/22, 16-53%) |  |
|  | E | 60% (18/30, 42-76%) |  |
|  | F | 0% (0/3, 0-71%) |  |
|  | G | 72% (13/18, 48-88%) |  |
| Cape Town  (outbreak) | NA | NA | 67% (8/12, 38-87%) |


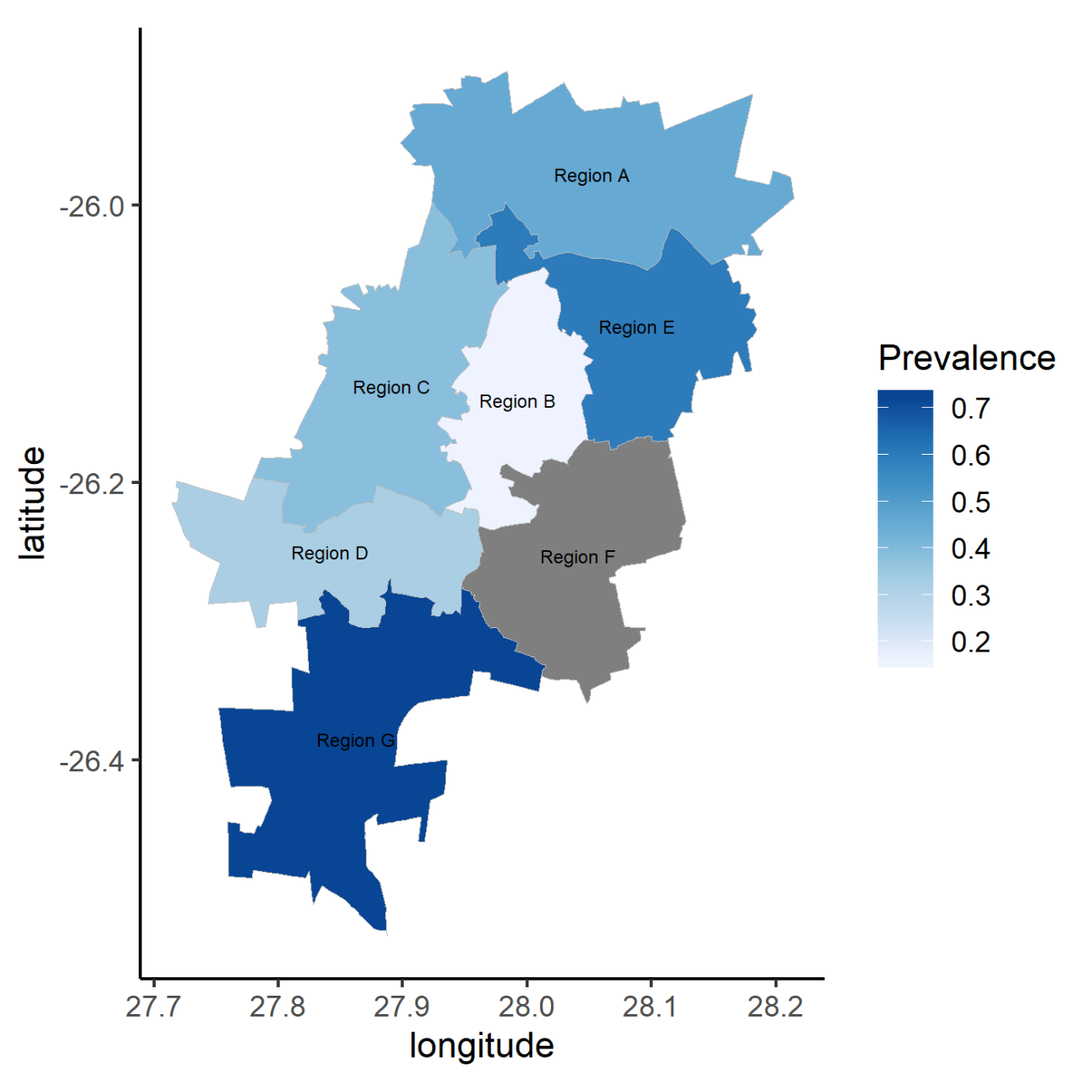


Figure S2: The spatial distribution of regional prevalence across the seven administrative regions of the City of Johannesburg. Region F, where only three animals were tested is excluded from the analysis

Reference:

1. Naidoo K, Moseley M, McCarthy K, Chingonzoh R, Lawrence C, Setshedi GM, et al. Fatal Rodentborne Leptospirosis in Prison Inmates, South Africa, 2015. Emerg Infect Dis. 2020;26(5). https://doi.org/10.3201/eid2605.191132
