# Supplementary material for: Multi-locus sequence analyses reveal a clonal L. borgpetersenii genotype in a heterogeneous invasive Rattus spp. community across the City of Johannesburg, South Africa
Source: Parasit Vectors. 2020 Nov 11;13:570. doi: 10.1186/s13071-020-04444-0 (PMC7659165; doi:10.1186/s13071-020-04444-0)
Supplement: Supplementary file 3 — Additional file 3: Figure S3. Maximum clade credibility tree based on L. borgpetersenii lfb1 sequences (167 bp) implemented using the Jukes-Cantor evolutionary model. Figure S4. Maximum clade credibility tree based on L. borgpetersenii secY sequences (433bp) implemented using the Jukes-Cantor evolutionary model. Figure S5. Maximum clade credibility tree based on L. borgpetersenii lipL41 sequences (594 bp) implemented using the Jukes-Cantor evolutionary model. Figure S6. Maximum clade credibility tree based on L. interrogans lfb1 sequences (261 bp) implemented using the Jukes-Cantor substitution model. Figure S7. Maximum clade credibility tree based on L. interrogans secY sequences (433 bp) implemented using the Hasegawa-Kishino-Yano evolutionary model (4) substitution model. Figure S8. Maximum clade credibility tree based on L. interrogans MST1 sequences (174 bp) implemented using the Hasegawa-Kishino-Yano evolutionary model (4) substitution model. Figure S9. Maximum clade credibility tree based on L. interrogans MST3 sequences (220 bp) implemented using the Hasegawa-Kishino-Yano evolutionary model (4) with a gamma distribution (4 categories). Figure S10. Maximum clade credibility tree based on L. interrogans MST9 sequences (204 bp) implemented using the Jukes-Cantor evolutionary model. [file 13071_2020_4444_MOESM3_ESM.docx]

**Phylogenetic analyses of individual loci**

Phylogenetic analyses were implemented in BEAST v2.6.0 (1) using the most appropriate substitution models as determined Bayesian Information Criterion (BIC) by model test in MEGA7 (2) and linked clocks (strict) and trees. Single-locus analyses were run using a chain length of 1x10^7^ and sampled every 1x10^3^ runs with a burn-in of 10%. TRACER v1.7.1 (3) was used to verify that the effective sample size (ESS) was greater than 200 and TREEANNOTATOR v2.6.0 used to generate a maximum clade credibility tree using mean node heights annotated by posterior probabilities greater than 0.9.

Electropherograms of sequences generated in the course of this study were examined by eye and trimmed for quality. *Leptospira* reference sequences were obtained by querying sequences against the NCBI refseq_genome database using the BLASTn algorithm limited to *Leptospira* (taxid 171) belonging to the two species (*L. interrogans* and *L. borpetersenii*) identified in this study. Aligned BLAST hits for each locus were linked by biosample and representative sequences for each *Leptospira* species and serovar combination selected as reference sequences.

1. ***L. borgpetersenii***


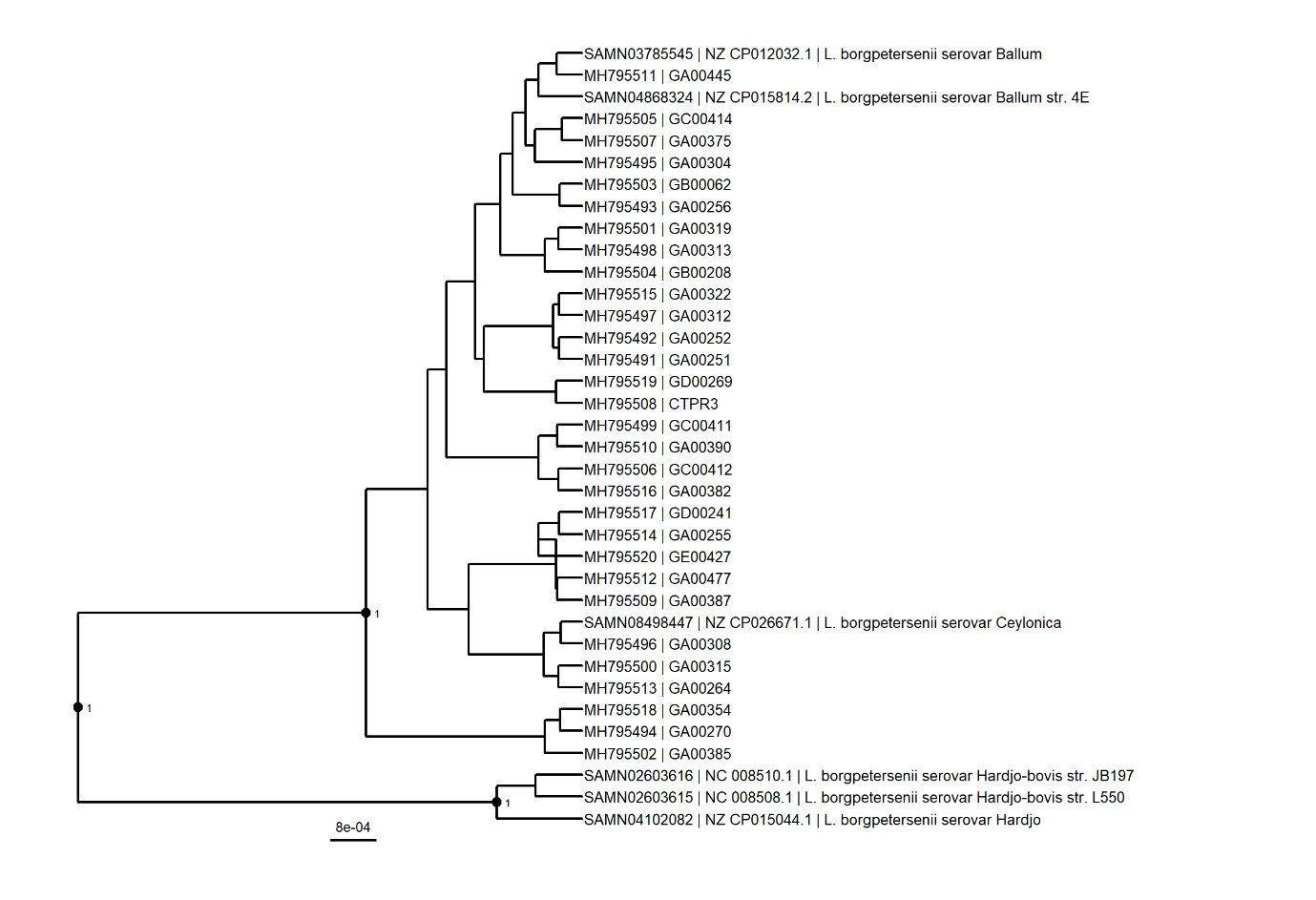


Figure S3: Maximum clade credibility tree based on *L. borgpetersenii* *lfb1* sequences (167bp) implemented using the Jukes-Cantor evolutionary model. Nodes with posterior support greater than 0.9 are shown. Reference sequences are labelled by biosample, sequence accession, and *Leptospira* strain and sequences from this study labelled by accession and sample code. Sample codes beginning with “G” are from the Johannesburg study site and codes beginning with “C” from Cape Town. Although 37 *lfb1* products were sequenced to confirm the *Leptospira* species identification inferred from melt curve analysis, eight sequences were of insufficient quality for phylogenetic analysis and were excluded


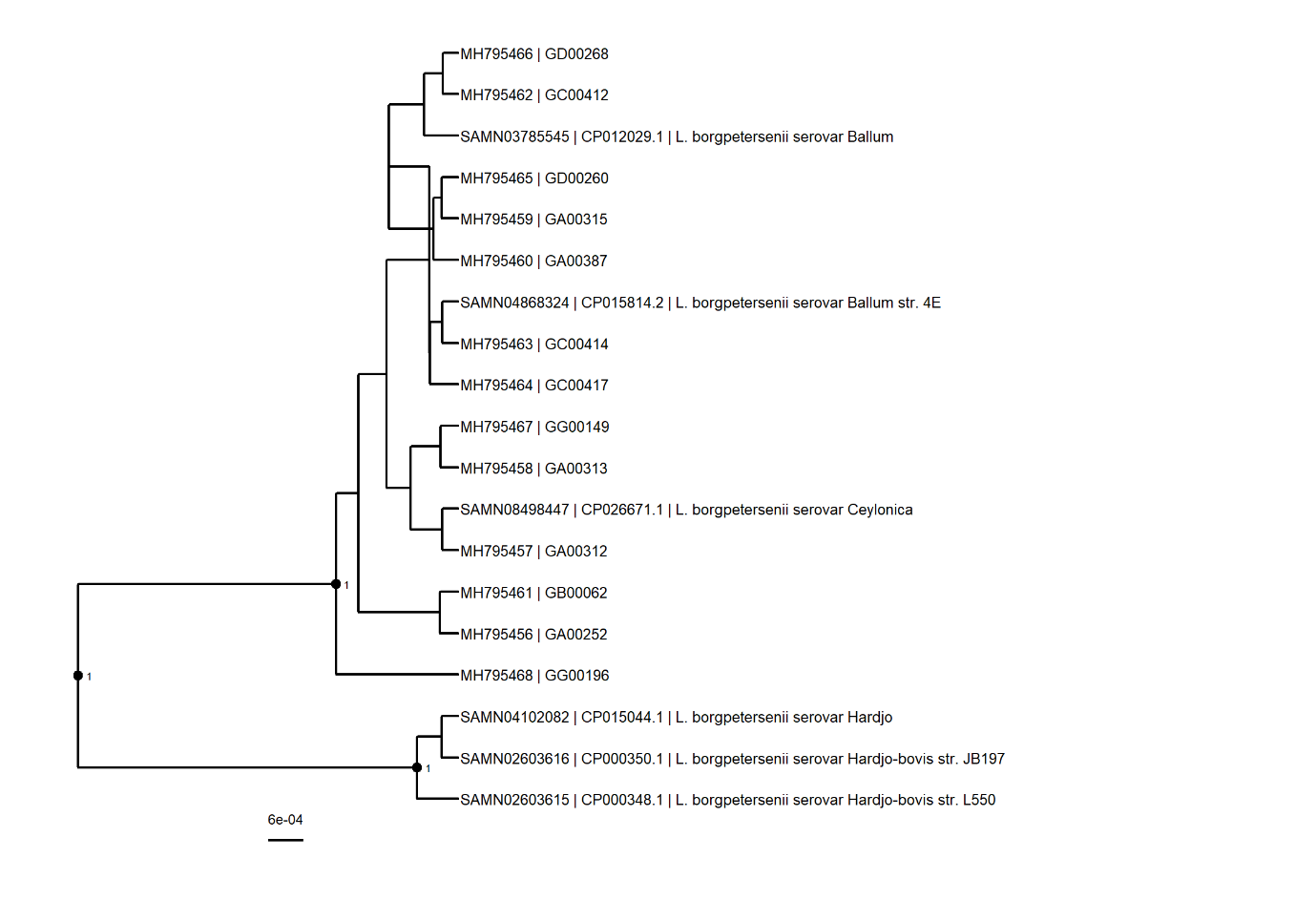


Figure S4: Maximum clade credibility tree based on *L. borgpetersenii* *secY* sequences (433bp) implemented using the Jukes-Cantor evolutionary model. Nodes with posterior support greater than 0.9 are shown. Reference sequences are labelled by biosample, sequence accession, and *Leptospira* strain and sequences from this study labelled by accession and sample code


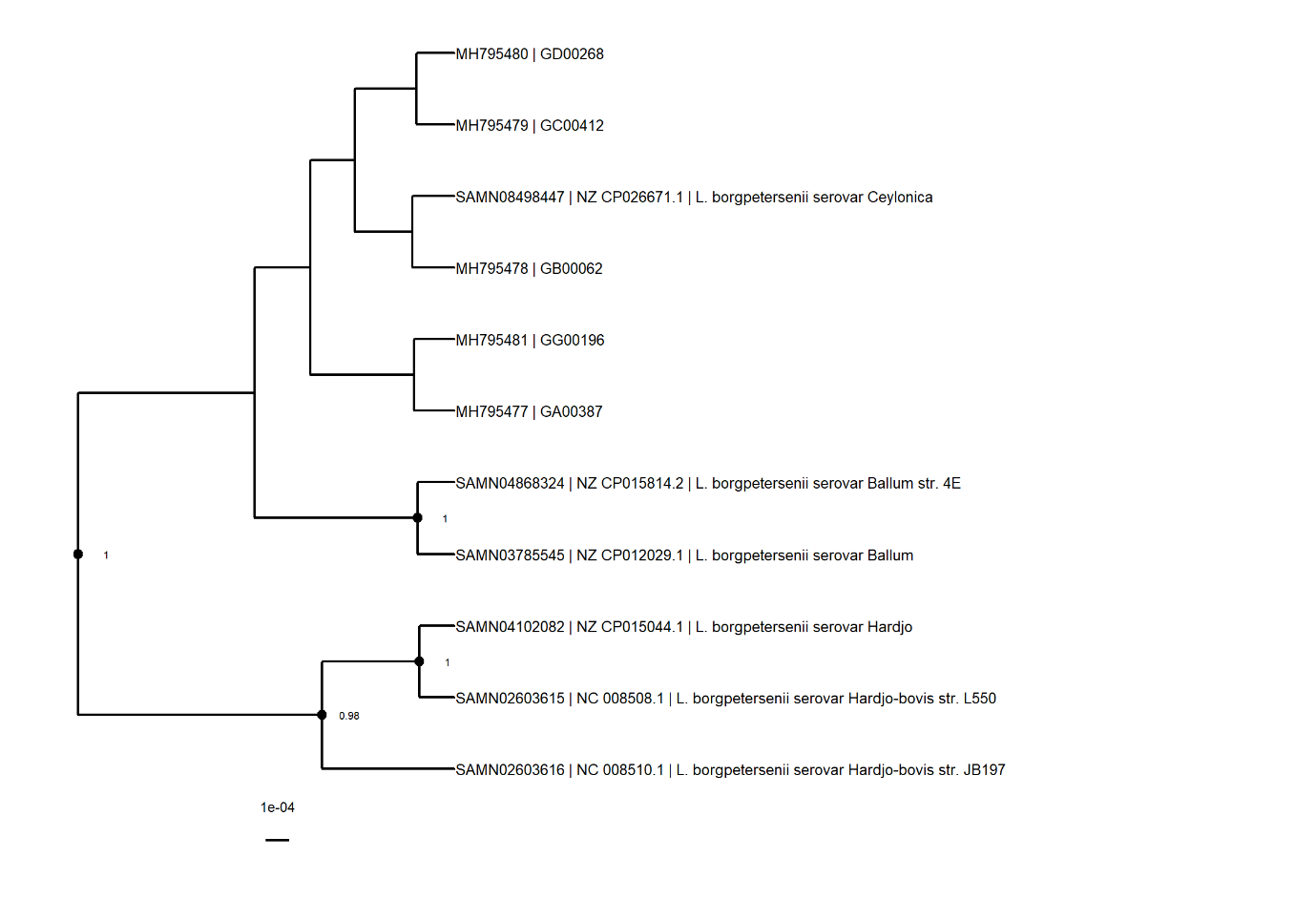


Figure S5: Maximum clade credibility tree based on *L. borgpetersenii* *lipL41* sequences (594bp) implemented using the Jukes-Cantor evolutionary model. Nodes with posterior support greater than 0.9 are shown. Reference sequences are labelled by biosample, sequence accession, and *Leptospira* strain and sequences from this study labelled by accession and sample code

1. ***L. interrogans***


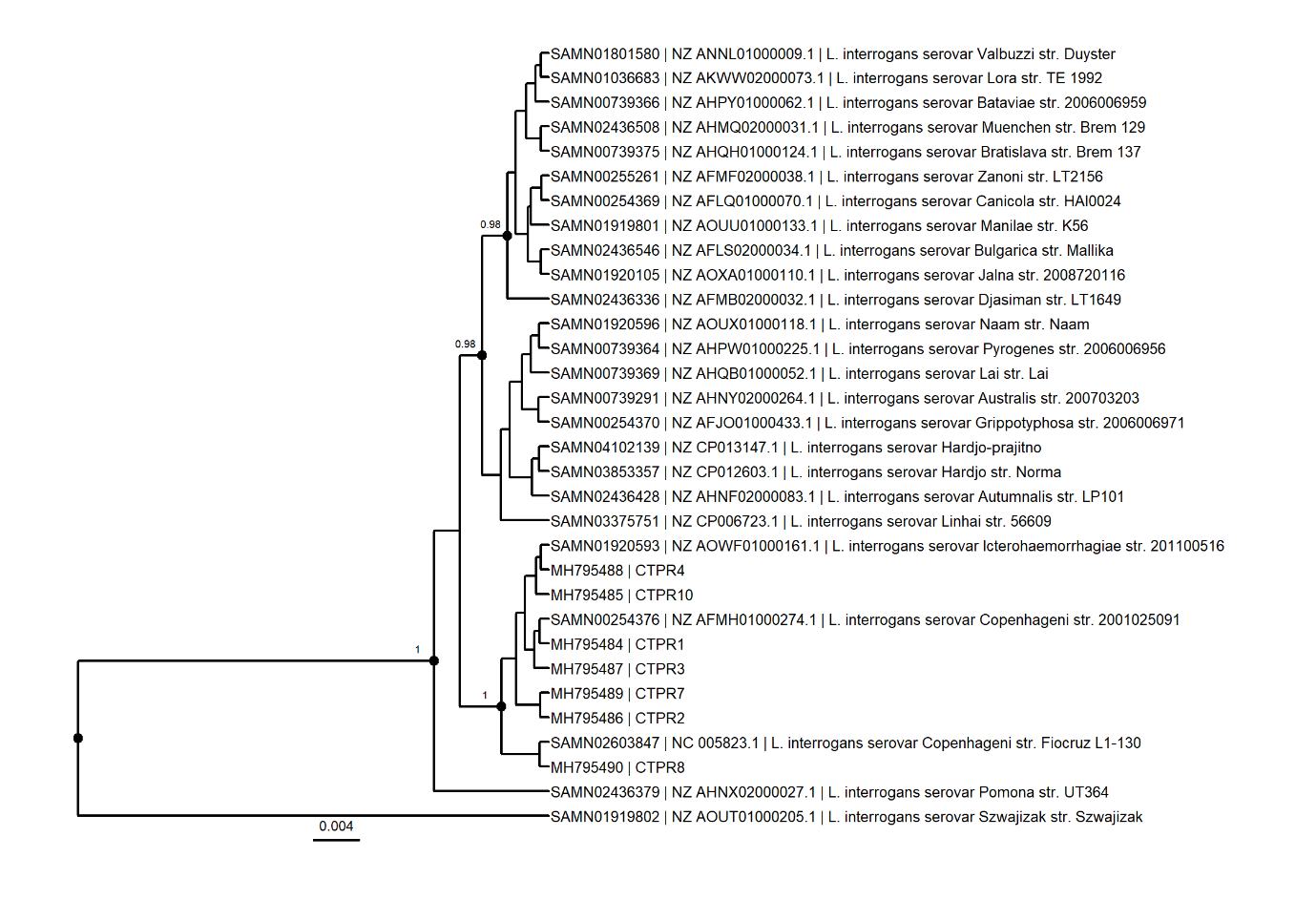


Figure S6: Maximum clade credibility tree based on *L. interrogans* *lfb1* sequences (261bp) implemented using the Jukes-Cantor substitution model. Nodes with posterior support greater than 0.9 are shown. Reference sequences are labelled by biosample, sequence accession, and *Leptospira* strain and sequences from this study labelled by accession and sample code


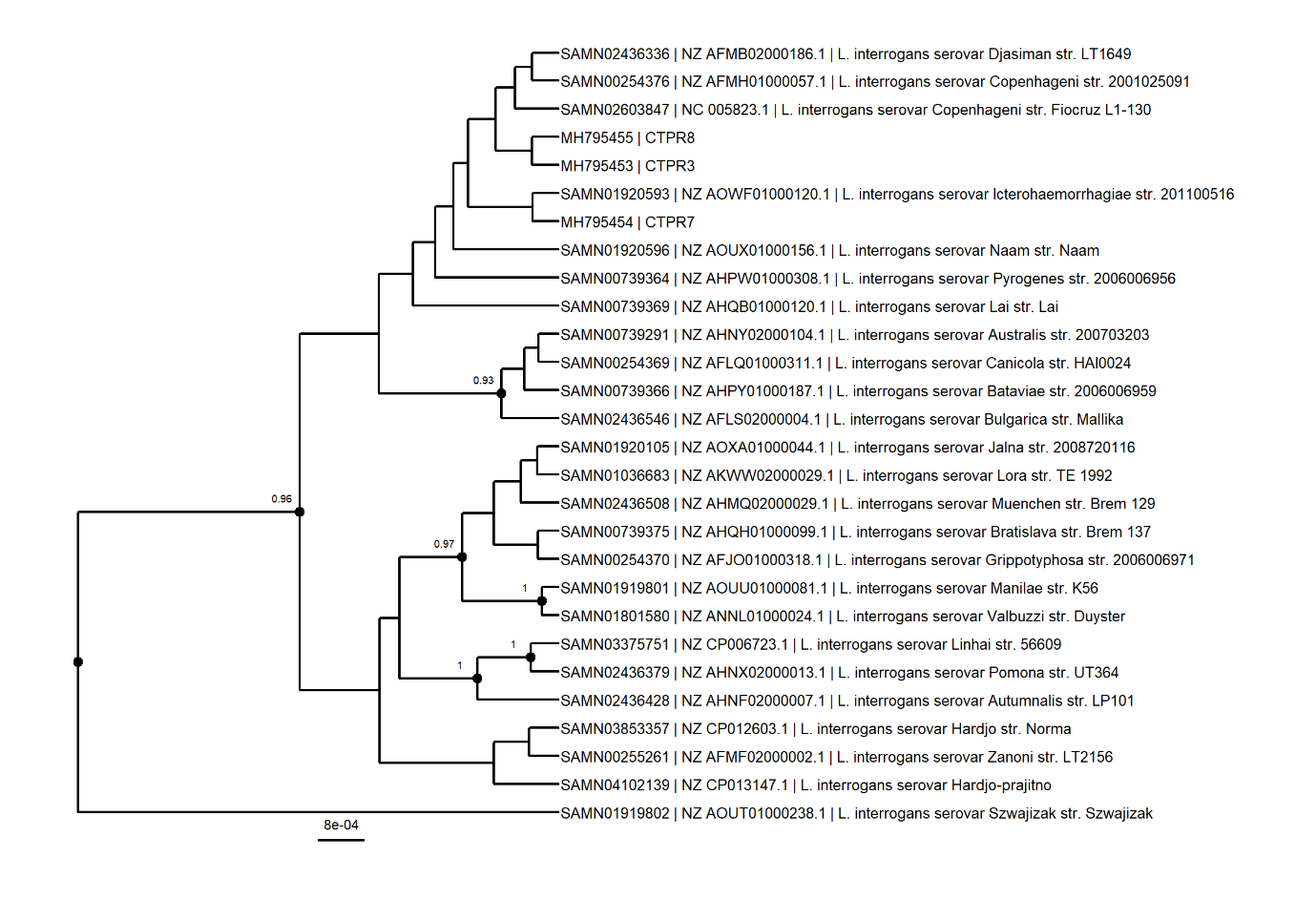


Figure S7: Maximum clade credibility tree based on *L. interrogans* *secY* sequences (433bp) implemented using the Hasegawa-Kishino-Yano evolutionary model (4) substitution model. Nodes with posterior support greater than 0.9 are shown. Reference sequences are labelled by biosample, sequence accession, and *Leptospira* strain and sequences from this study labelled by accession and sample code


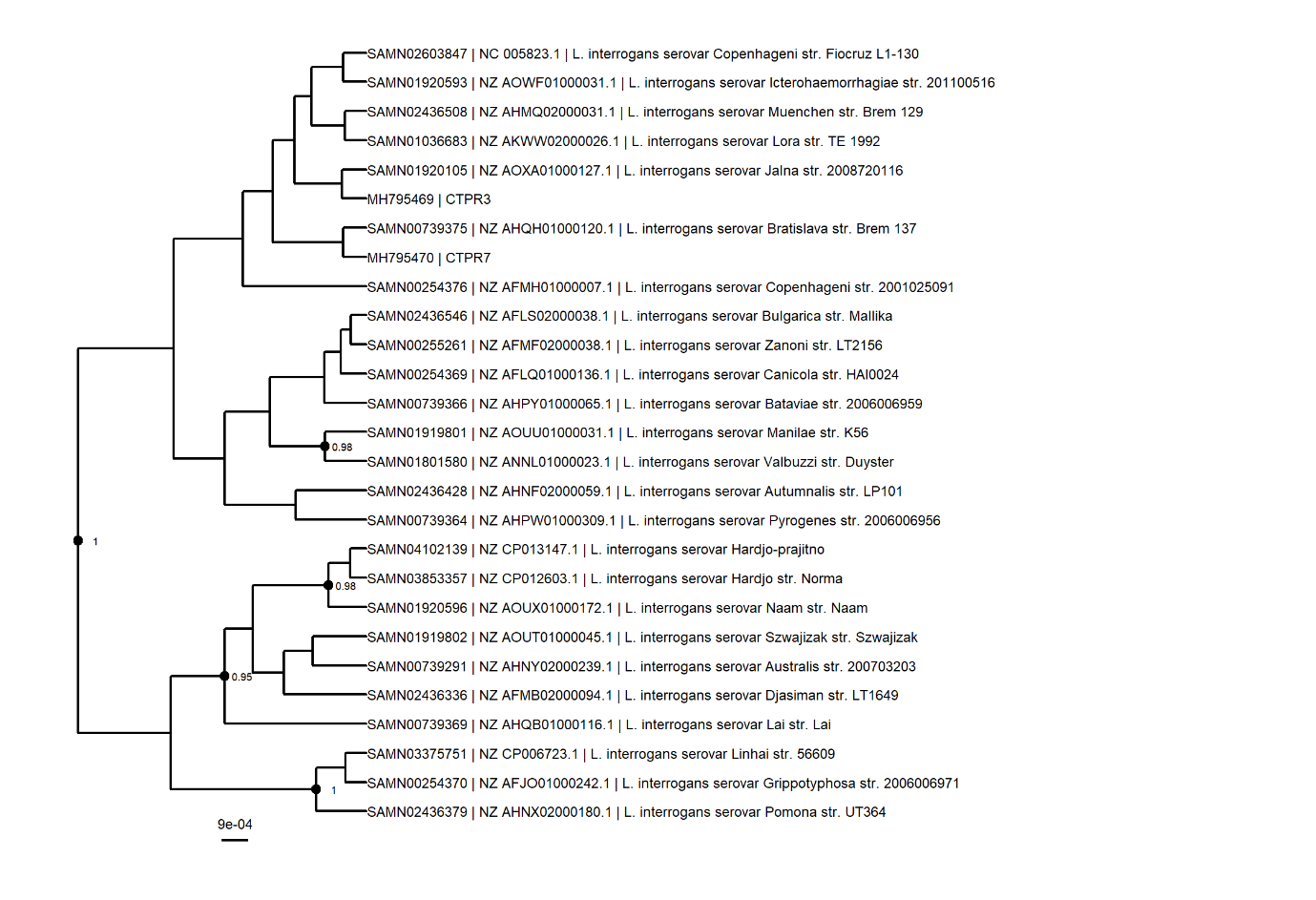


Figure S8: Maximum clade credibility tree based on *L. interrogans* MST1 sequences (174bp) implemented using the Hasegawa-Kishino-Yano evolutionary model (4) substitution model. Nodes with posterior support greater than 0.9 are shown. Reference sequences are labelled by biosample, sequence accession, and *Leptospira* strain and sequences from this study labelled by accession and sample code


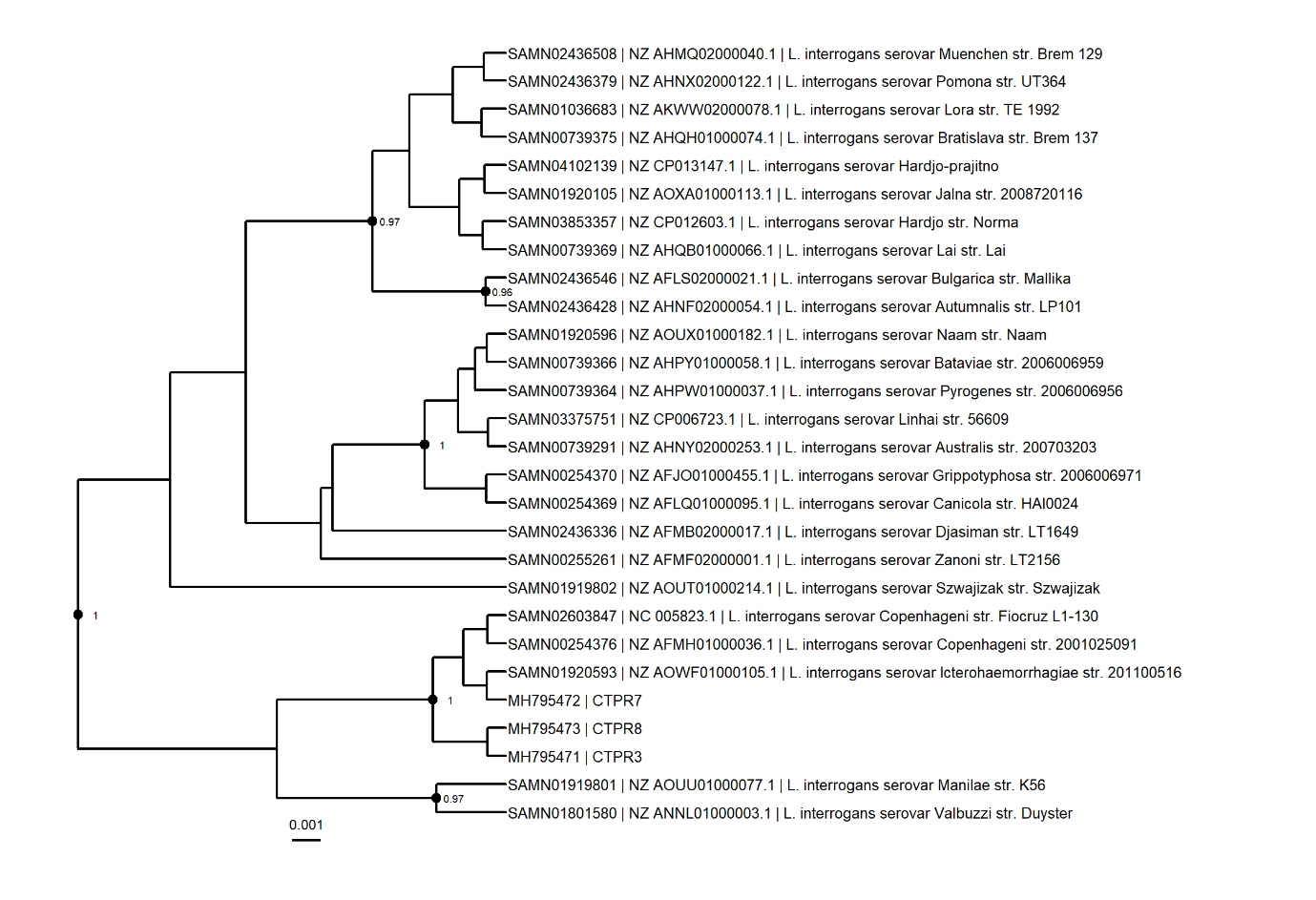


Figure S9: Maximum clade credibility tree based on *L. interrogans* MST3 sequences (220bp) implemented using the Hasegawa-Kishino-Yano evolutionary model (4) with a gamma distribution (4 categories). Nodes with posterior support greater than 0.9 are shown. Reference sequences are labelled by biosample, sequence accession, and *Leptospira* strain and sequences from this study labelled by accession and sample code


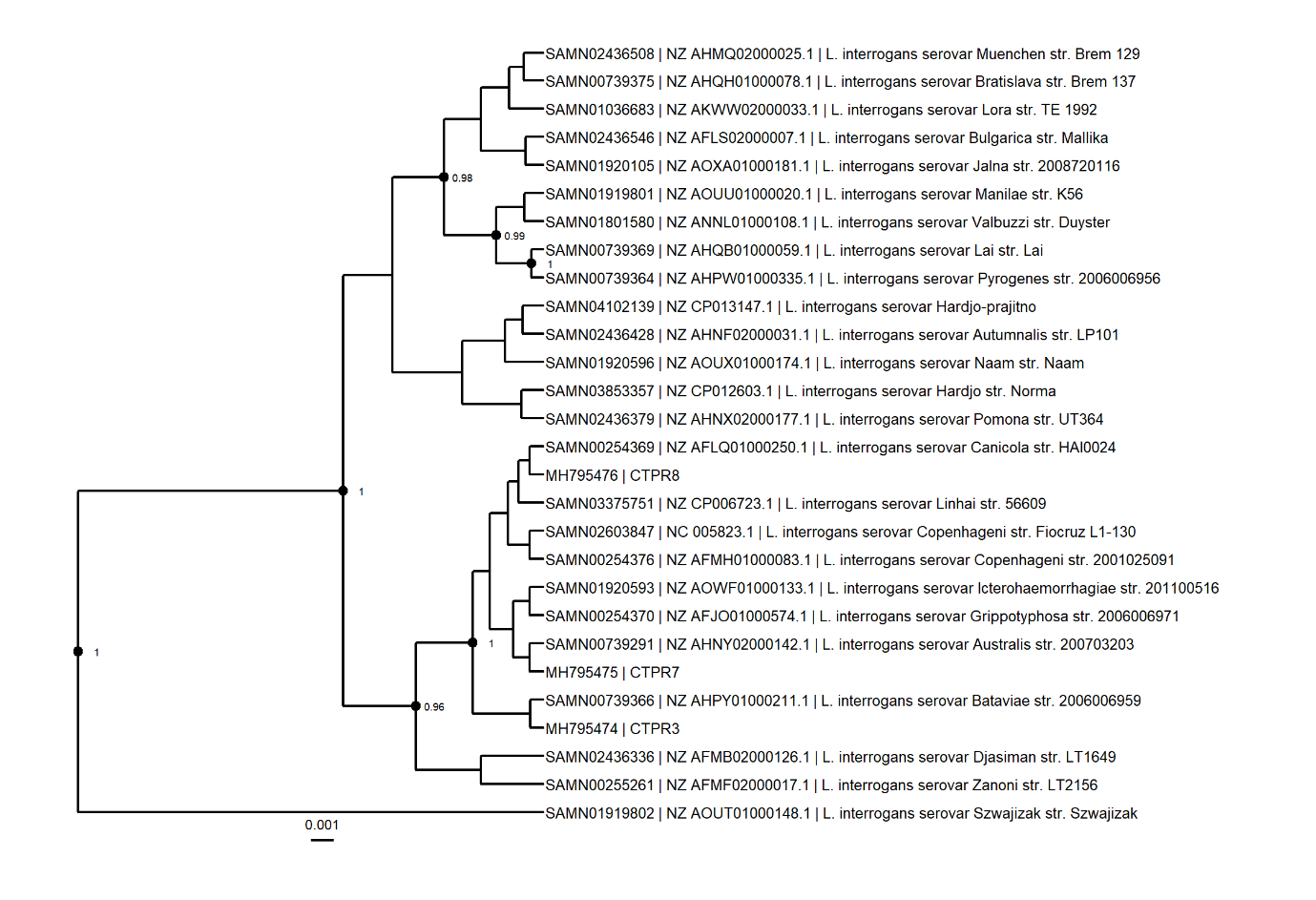


Figure S10: Maximum clade credibility tree based on *L. interrogans* MST9 sequences (204bp) implemented using the Jukes-Cantor evolutionary model. Nodes with posterior support greater than 0.9 are shown. Reference sequences are labelled by biosample, sequence accession, and *Leptospira* strain and sequences from this study labelled by accession and sample code

References:

1. Bouckaert R, Heled J, Kühnert D, Vaughan T, Wu C-H, Xie D, et al. BEAST 2: a software platform for Bayesian evolutionary analysis. PLoS Comput Biol [Internet]. 2014 Apr [cited 2014 Jul 11];10(4):e1003537. Available from: http://www.pubmedcentral.nih.gov/articlerender.fcgi?artid=3985171&tool=pmcentrez&rendertype=abstract

2. Kumar S, Stecher G, Tamura K. MEGA7: Molecular Evolutionary Genetics Analysis Version 7.0 for Bigger Datasets. Mol Biol Evol. 2016;33(7):1870–4.

3. Rambaut A, Drummond AJ, Xie D, Baele G, Suchard MA. Posterior summarization in Bayesian phylogenetics using Tracer 1.7. Syst Biol. 2018;67(5):901–4.

4. Hasegawa M, Kishino H, Yano T aki. Dating of the human-ape splitting by a molecular clock of mitochondrial DNA. J Mol Evol. 1985;22(2):160–74.
